# Tourism-associated short-term variability of large microplastics on a tropical sandy beach in the Southern Gulf of Mexico

**DOI:** 10.1007/s10661-026-15678-3

**Published:** 2026-07-21

**Authors:** Moisés Vallejo Gomez, María del Carmen Cuevas Díaz, Michele Arienzo, Claudia Cucolo, Luciano Ferrara, Marco Trifuoggi, José Aurelio Sosa Olivier, José Ramón Laines Canepa

**Affiliations:** 1 División de Ciencias Biológicas, Universidad Juárez Autónoma de Tabasco, Centro, Tabasco México; 2 Facultad de Ciencias Químicas, Universidad Veracruzana, Coatzacoalcos, Veracruz México; 3 Dipartimento Di Scienze Della Terra, Dell’Ambiente E Delle Risorse, Università Degli Studi Di Napoli Federico II, Complesso Universitario Di Monte Sant’Angelo, Via Cintia 26, 80126 Naples, Italy; 4 Dipartimento Di Scienze Chimiche, Università Degli Studi Di Napoli Federico II, Complesso Universitario Di Monte Sant’Angelo, Via Cintia 26, 80126 Naples, Italy

**Keywords:** Beach litter, FTIR identification, Polymer composition, Coastal monitoring, Visitor pressure, Sediment pollution

## Abstract

Tourism-associated coastal activities represent an important source of plastic-derived debris in tropical environments; however, baseline information on large microplastics (> 0.84 mm) remains limited in the Southern Gulf of Mexico. This study evaluated short-term variability in the composition and abundance of confirmed large microplastics in beach sediments from Playa Azul, Centla, Tabasco, comparing two contrasting visitation periods. Two 1-week monitoring campaigns representing low- and high-visitation conditions were conducted to assess changes in daily beach debris generation and large-microplastic characteristics. Results showed a marked increase in total beach debris during the high-visitation period, reaching values approximately 25-fold higher than those recorded during the low-visitation period. In contrast, confirmed large-microplastic abundance showed only limited short-term variation, while morphological composition differed between campaigns. Foams and fragments predominated during the low-visitation period, whereas films and fibres increased during the high-visitation period. Carbonaceous particles were recorded separately as non-plastic anthropogenic residues because their polymeric origin was not confirmed by FTIR. Polymer characterization using Fourier-transform infrared spectroscopy (FTIR) revealed high spectral matches for high-impact polystyrene (HIPS) in foams (95–98%), high-density polyethylene (HDPE) in fragments (90–95%), and polyethylene (PE) in fibres (90–92%). The Microplastic Pollution Index (MPPI) was interpreted as a descriptive screening indicator rather than as a definitive long-term pollution classification. Overall, the findings provide a short-term baseline indicating that visitor pressure was clearly associated with increased total beach debris, whereas large-microplastic abundance and morphology require broader temporal and spatial replication before robust long-term or causal conclusions can be drawn.

## Introduction

Mexico generates substantial quantities of municipal solid waste, and plastic residues remain a priority concern because inadequate collection, leakage, and coastal transport can contribute to marine litter and microplastic formation. In recent decades, plastic debris has become a pressing environmental concern due to increased consumption, inadequate waste management practices, widespread dispersion, and long-term environmental persistence. Plastic production has expanded from 1.5 million metric tons in 1950 to 400.3 million metric tons in 2022 (Statista Research Department, 2025). Approximately 710 million tons of plastic waste enter aquatic and terrestrial ecosystems worldwide, contributing significantly to environmental pollution (Kibria et al., 2023; Ranjani et al., 2021). Marine debris generated by human activities includes a wide range of materials such as fishing nets, bottles, caps, and fragmented plastics of varying sizes. Plastics are commonly classified according to size as mega-plastics (MG; > 1 m), macro-plastics (MA; 25 mm–1 m), meso-plastics (ME; 5–25 mm), microplastics (MPs; 1 µm–5 mm), and nano-plastics (NP; < 1 µm) (Masura et al., 2015; Rodríguez-Seijo & Pereira, 2017). Up to 11% of discarded plastics become MPs and the transformation is driven by physical, chemical, and biological fragmentation processes (Laines Canepa et al., 2024). As MPs become smaller, they may cross biological barriers and pose risks to biodiversity and ecosystem functioning. Moreover, MPs may act as vectors for pathogens and co-contaminants, with subsequent release into living organisms (Bollaín Pastor & Vicente Agulló, 2019), highlighting the need for sustainable waste management strategies to mitigate their environmental impacts (Laines Canepa et al., 2024).

One major destination of plastic debris and MPs is sandy beaches that as a vital part of a coastal ecosystem, act as a major sink. These environments are particularly vulnerable during periods of high tourist influx, when increased human activity can intensify the accumulation and redistribution of anthropogenic debris. Consequently, beach environmental quality requires continuous monitoring to maintain ecological integrity and ensure safe recreational conditions. The evaluation of beach environmental quality involves the assessment of physical, biological, and social parameters. This multidimensional concept is essential for guiding effective coastal management strategies, protecting visitor health, and supporting sustainable decision-making adapted to local environmental conditions (Botero Saltaren et al., 2013).

Recent reviews have highlighted the limited availability of studies addressing MP abundance and composition in beach sediments across Latin America (Mesquita et al., 2022). Despite accounting for approximately 4% of global plastic production and 8% of plastic consumption, Central American and Caribbean regions remain underrepresented in MP research (Kutralam-Muniasamy et al., 2020; Mesquita et al., 2022). This scientific gap underscores the vulnerability of Latin American coastal environments to plastic pollution and emphasizes the urgent need for baseline monitoring data. In Mexico, inadequate waste management practices have contributed to the increasing presence of MPs in coastal and marine environments, particularly in areas with intense tourism or industrial activities (Vázquez-Morillas et al., 2023). This situation is especially relevant in Tabasco, a coastal state located in the Southern Gulf of Mexico, where mangrove systems and tourism-driven sandy beaches play a critical role in regional biodiversity and socio-economic development. Consequently, accurate assessments of plastic pollution in these coastal habitats are essential to support evidence-based environmental management and sustainable coastal planning.

The primary objective of this study was to quantify and characterize large MPs (> 0.84 mm) in beach sediments from Playa Azul, Villa Cuauhtémoc (Centla, Tabasco), located in the Southern Gulf of Mexico, and to compare their abundance and composition between two short-term periods with contrasting visitor pressure. Specifically, the study examined how low- and high-visitation periods influence MP abundance, composition, and beach environmental quality. The findings aim to provide baseline monitoring data for the regional coastline using a consistent sampling and identification framework, facilitating future regional comparisons. This will help draw an integrated plastic waste management model and policy responses for conservation actions and amelioration of the overall environmental quality of the region, lowering MP exposure and environmental risk in humans and biota.

## Materials and methods

### The study area

One sandy beach located in the coastal zone of Tabasco was selected based on accessibility criteria and international coastal monitoring recommendations (GESAMP, 2019), Fig. 1. The study site, Playa Azul, Villa Cuauhtémoc (Centla, Tabasco, Mexico), is situated at 18° 32′ 15″ N and 92° 42′ 22″ W in the Southern Gulf of Mexico, approximately 6 km west of Frontera and near the mouth of the Grijalva River. The proximity to this major fluvial system allows assessment of potential inland contributions to coastal plastic inputs and supports future integrated monitoring of water, sediments, and biota. Frontera represents one of the main ports in the region, characterized by maritime and commercial activity as well as tourism-driven coastal use. Playa Azul ranks third among the seven officially recognized beaches in Tabasco State.

**Fig. 1:**
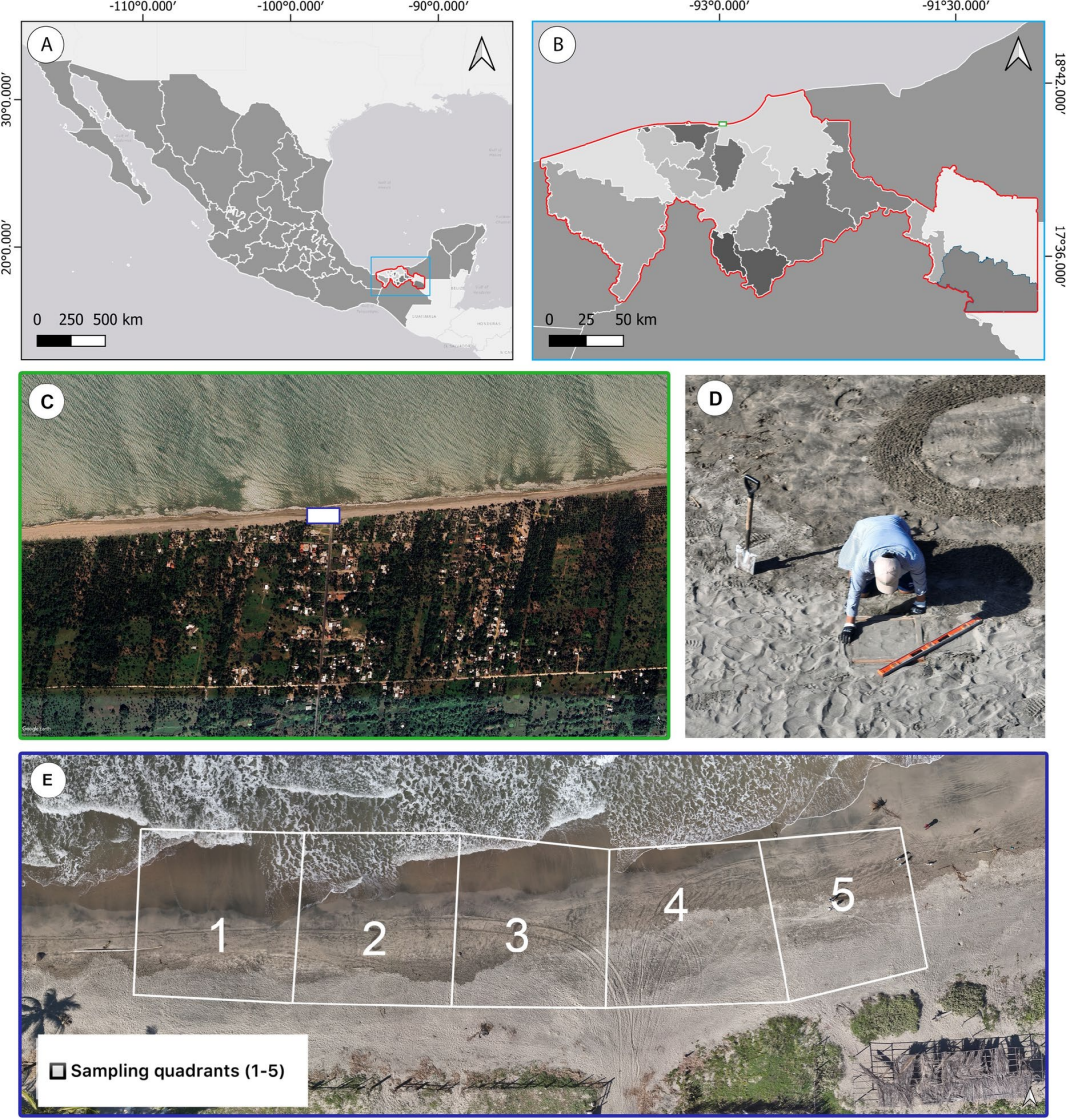
Geographic location of the study area: **A** Mexico, **B** Tabasco State, where shaded areas indicate municipal boundaries and the study municipality, **C** Playa Azul, Villa Cuauhtémoc, Centla, **D** field sampling activities, and **E** aerial view of the sampling sectors. Aerial imagery source: Google Earth/Maxar Technologies, accessed 2026

The region presents a tropical climate with a mean annual temperature of 29 °C, potentially reaching 31 °C during warmer periods. Prevailing winds are predominantly easterly, with speeds ranging from 10 to 15 km/h and reaching up to 18 km/h during peak daytime heating. Relative humidity ranges from 50 to 70% during daytime and may increase to 85% at night (CONAGUA, 2025).

### Sampling procedure

Two sampling campaigns were conducted to represent contrasting tourism scenarios: low-visitation period (15–21 February) and high-visitation period (12–19 April). One day prior to initiating daily monitoring during each campaign, the beach was cleaned (time zero), collecting 68.78 kg (low-visitation period) and 76.46 kg (high-visitation period) of solid marine debris. This baseline cleaning allowed subsequent comparison of daily waste accumulation under natural tourist activity conditions.

Daily per capita waste generation was calculated using:

1 $$Mv=\frac{M}{V}$$

where Mv represents the per capita debris generation (kg visitor⁻^1^), *M* is the total mass of debris collected per day (kg day⁻^1^), and *V* is the number of visitors recorded during the sampling day. The parameter Mv was calculated as the ratio between the total daily mass of debris collected (M) and the number of visitors recorded during the sampling day (V).

Microplastics (MPs) were sampled following CEGESTI (2022) guidelines, which were used as an operational guide for beach solid-waste sampling rather than as a globally standardized microplastic protocol. Because its use in peer-reviewed microplastic studies remains limited, the microplastic handling and interpretation procedures were cross-referenced with internationally recognized monitoring recommendations, including GESAMP (2019) guidelines and NOAA laboratory procedures for microplastic analysis (Masura et al., 2015).

Field sampling was conducted using a standardized transect–quadrat approach. A transect measuring 20 m in width and 100 m in length was established parallel to the upper high-tide line (Fig. 1).

Three non-adjacent sectors were selected for sampling: field sectors 1, 3, and 5. For analytical reporting, these selected sectors were coded as Q1, Q2, and Q3, respectively. Within each selected sector, five 0.5 m × 0.5 m quadrats were sampled at the four corners and centre, to a depth of 1 cm, avoiding disturbance of the surrounding sediment. This resulted in 15 quadrats per campaign and a total sampled surface area of 3.75 m^2^ per campaign. Samples were placed in labelled bags for laboratory processing.

In the laboratory, samples were weighed, sun-dried for 5 h, and sieved according to the ASTM E11 Standard Test Sieve Series at mesh sizes of 4.76, 2.00, 1.00, and 0.84 mm. Particles retained on each sieve were collected and stored in labelled containers, while visible organic matter was manually removed.

### Analytical procedure

Sediment residues were placed in Petri dishes and treated with 50 mL of 35% H_2_O_2_ at 40 °C for 48 h to digest residual organic matter. After digestion, samples were rinsed thoroughly with distilled water. Subsequently, three drops of 0.5 N HCl were added to remove calcareous materials. Samples were rinsed again to eliminate acidic residues and dried at 60 °C until constant weight, yielding clean, dry material for further analysis.

### Morphoscopic characterization

Dried particles were examined under a Velab VE-153G stereoscopic microscope equipped with an LCD screen. Particles were individually separated using ultra-fine tweezers, measured, counted, and recorded. Confirmed large microplastic particles were classified according to morphological categories following Purca and Henostroza (2017): foam, fragment, film, fibres, and others. Carbonaceous particles were recorded separately as non-plastic anthropogenic residues because their polymeric origin was not confirmed by FTIR.

### Quantification of MP impact

Microplastic counts and particle densities (particles kg^−1^ dry sediment) were used for data processing and analysis. Two environmental indices were used to evaluate the effects of MPs.

The first index is the MP Pollution Index (MPPI), calculated as particle density per unit surface area:

2 $$\mathrm{MPPI}=\frac{\mathrm{Total}\;\mathrm{number}\;\mathrm{of}\;\mathrm{MPs}}{\mathrm{Surveyed}\;\mathrm{area}}$$

MPPI was calculated as the number of confirmed large microplastic particles divided by the total sampled surface area (3.75 m^2^ per campaign) and expressed as particles m^−2^. Mean ± standard deviation values were calculated using quadrats as replicate sampling units. Given the limited temporal replication, MPPI and CMPI were used as descriptive screening tools to support comparison between campaigns, not as definitive indicators of long-term beach pollution status. MPPI classes were defined as follows: 0–2 (low abundance or absence), 2–5 (low to moderate presence), 5–15 (moderate abundance), 15–25 (high presence), and > 25 (very high load) (Rangel Buitrago et al., 2021).

To evaluate the relative contribution of particle morphology, the coefficient of MP impact (CMPI) was calculated as:

3 $$\mathrm{CMPI}=\frac{\mathrm{Number}\;\mathrm{of}\;\mathrm{MPs}\;\mathrm{of}\;\mathrm a\;\mathrm{given}\;\mathrm{morphological}\;\mathrm{class}}{\mathrm{Total}\;\mathrm{numbers}\;\mathrm{of}\;\mathrm{MPs}}$$

This index expresses the proportion of a given morphological class relative to total MPs. Pollution risk categories were defined as follows: 0.0001–0.1 (minimum), 0.11–0.5 (average), 0.51–0.8 (maximum), and 0.81–1.0 (extreme) (Pervez et al., 2023).

### Fourier-transform infrared spectroscopy (FTIR)

Polymer identification was conducted using Fourier-transform infrared spectroscopy (FTIR) with a Perkin Elmer Frontier FT-IR/FIR spectrometer equipped with a universal attenuated total reflectance (UATR) accessory. Spectra were compared with reference polymer libraries for identification.

Samples were handled on paper with security fibres to minimize external contamination and cleaned with acetone prior to analysis. FTIR was performed on a representative subset of particles selected to cover the dominant morphologies, campaigns, and sampling sectors. Particles were selected based on morphology, visual integrity, and sufficient surface area for ATR contact. Assignments were accepted when spectral matches were ≥ 70% and diagnostic absorption bands were visually consistent with the reference spectrum. Spectra with ambiguous matches, low signal-to-noise ratios, or no diagnostic polymer bands were classified as unidentified and were not used to confirm polymer composition.

Statistical analyses were performed using STATISTICA v.5 (StatSoft Inc., Tulsa, OK, USA). Pearson correlation and linear regression were applied to evaluate the association between daily debris mass (*M*) and visitor numbers (*V*) during the high-visitation period.

## Results and discussion

### Quantification of marine debris

The daily mass of plastic debris generated during the low-visitation period did not show any significant temporal trend, with *M* values ranging from 0.04 kg day^−1^ on day 7 to 0.82 kg day^−1^ on day 6. Similarly, no temporal trend was observed in the number of visitors (*V*), which, except for day 1 (*V* = 200), remained low and varied between 3 and 40 visitors. Because of the high variability in both daily plastic generation and visitor numbers, Mv values were highly heterogeneous, ranging from 0.001 to 0.273 kg visitor^−1^.

In contrast, *M* values during the high-visitation period were substantially greater, with a mean value approximately 25 times higher than that observed during the low-visitation period (10.16 vs. 0.41 kg day^−1^). Moreover, *M* showed a clear temporal trend, increasing from 2.12 kg day^−^1 on day 1 to 19.68 kg day^−1^ on day 7, with a cumulative production of 71.1 kg (Table 1). A similar trend was observed for visitor numbers, which increased from 21 visitors on day 1 to 330 visitors on day 7, reaching a cumulative total of 1035 visitors.

**Table 1:** Daily production of marine debris (*M*, kg day^−1^), number of visitors (*V*), and per capita debris generation (Mv, kg visitor^−1^) during the low- and high-visitation periods

Time	Low-visitation period			High-visitation period		
	*M*	*V*	Mv	*M*	*V*	Mv
	kg day^−1^		kg visitor^−1^	kg day^−1^		kg visitor^−1^
1	0.49	200	0.002	2.12	21	0.101
2	0.54	9	0.060	3.16	24	0.132
3	0.17	25	0.007	2.90	30	0.097
4	0.09	29	0.003	4.22	88	0.048
5	0.74	3	0.247	20.64	218	0.095
6	0.82	3	0.273	18.38	324	0.057
7	0.04	40	0.001	19.68	330	0.060
Mean	0.41 ± 0.32	44 ± 70	0.085 ± 0.122	10.16 ± 8.85	148 ± 140	0.084 ± 0.030
Total	2.89	309		71.10	1035	

Thus, data from the high-visitation period showed a strong linear relationship between daily debris production and visitor numbers (*R*^2^ = 0.88, *n* = 7). The marked differences in both *M* and *V* between the two sampling periods offset each other, resulting in nearly identical mean Mv values (0.085 kg visitor^−1^) for both periods. These results indicate a strong association between visitor numbers and daily beach debris mass during the high-visitation period at Playa Azul.

Although the absolute debris mass differed markedly between visitation periods, the mean per capita generation (Mv ≈ 0.08 kg visitor^−1^) remained comparable (Table 1), indicating proportional scaling between tourism intensity and waste production.

### MP morphological composition

**Fig. 2:**
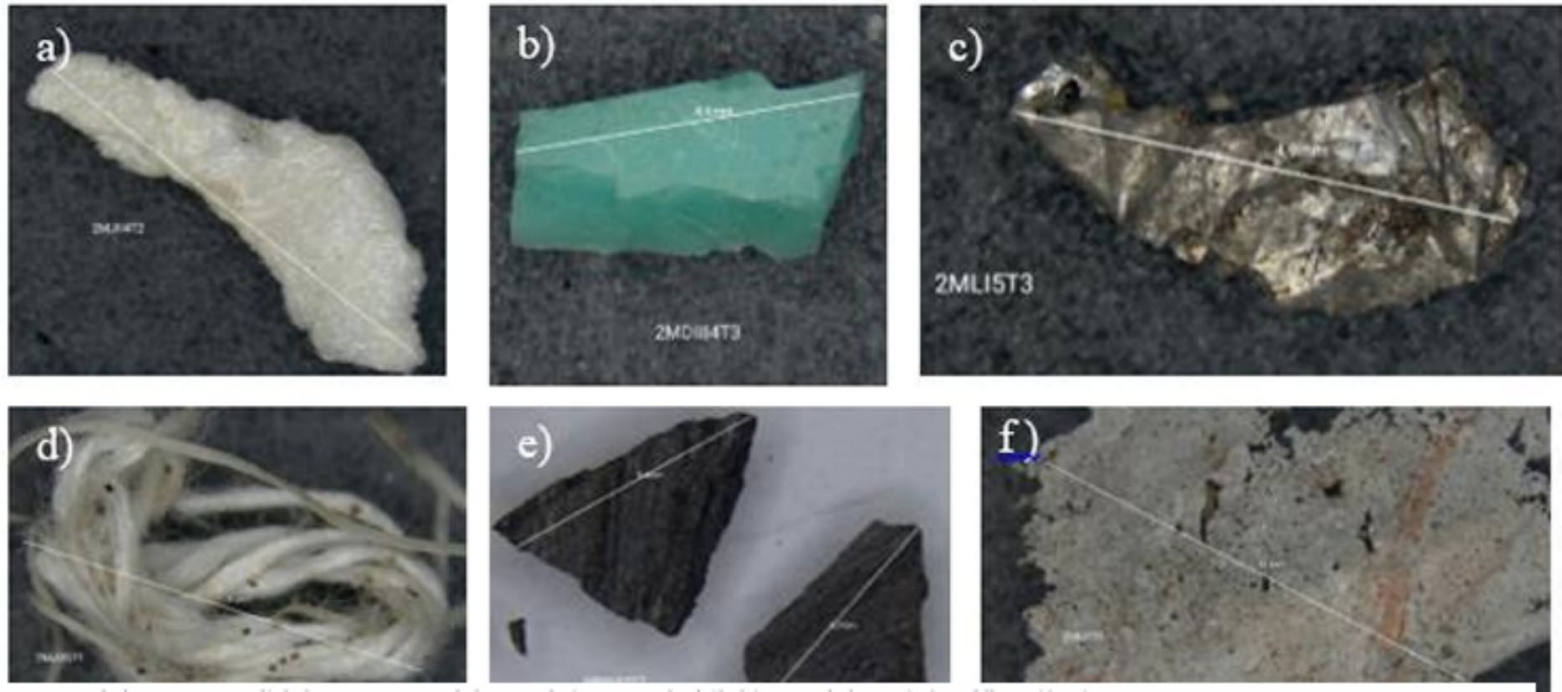
Microscopy identification of particle morphologies: **a** foam, **b** fragment, **c** film, **d** fibre, **e** carbonaceous particle recorded separately because its polymeric origin was not confirmed, and **f** others. White lines indicate the measurement axes used for particle size determination during morphoscopic characterization

The output of microscopy assessment is shown in Fig. 2. Confirmed large microplastics were classified into five morphological categories: foam, fragment, film, fibre, and others. Carbonaceous particles were recorded and illustrated separately because their polymeric origin was not confirmed by FTIR, and therefore, they were excluded from confirmed microplastic counts.

**Fig. 3:**
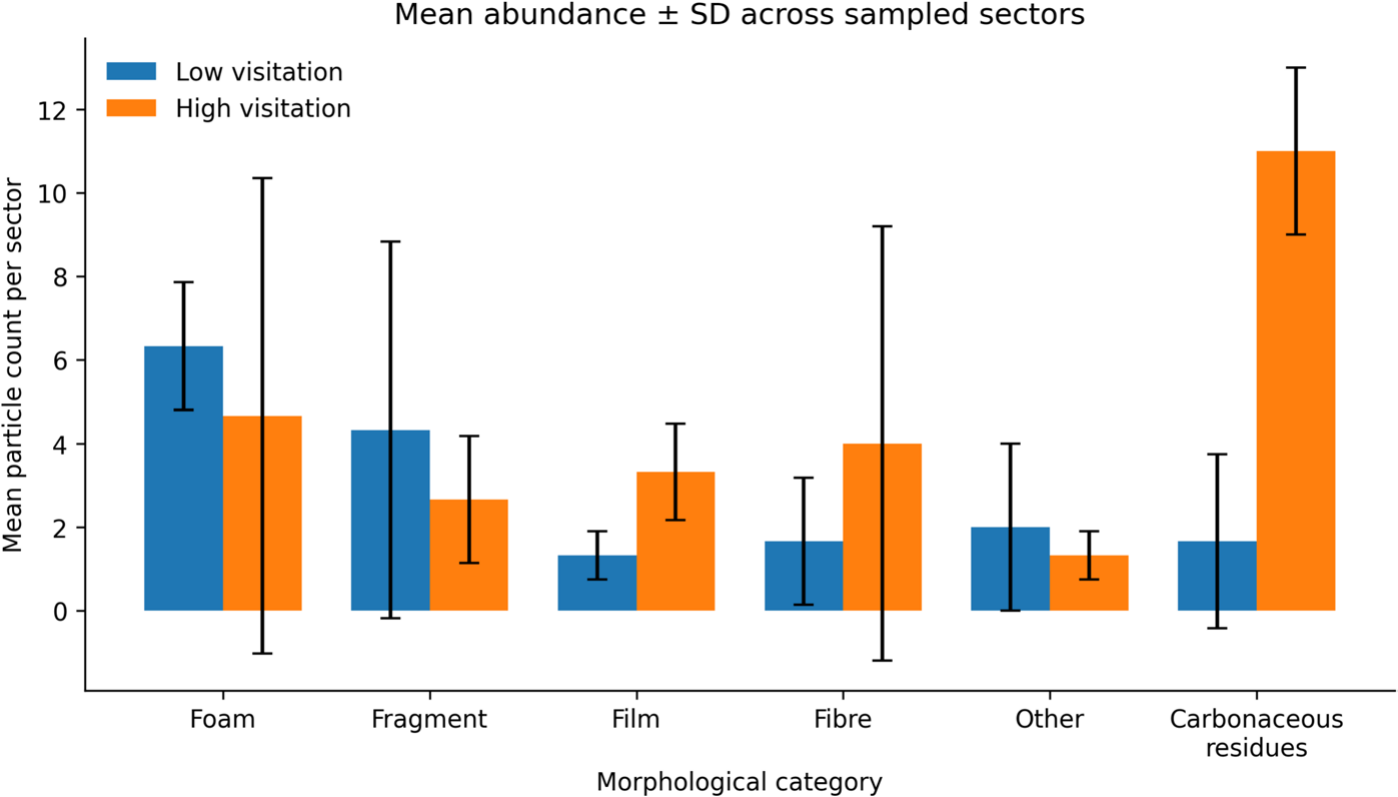
Mean abundance ± standard deviation of confirmed large microplastic morphologies and separately recorded carbonaceous residues across sampled sectors during low- and high-visitation periods at Playa Azul. Carbonaceous residues were not included in confirmed microplastic counts or CMPI calculations

The comparative analysis of particle morphologies among the three surveyed analytical sectors, Q1, Q2, and Q3, during the two contrasting visitation periods, in Fig. 3, showed that confirmed large microplastic abundance was low and spatially heterogeneous. Q1 had the lowest abundance of confirmed large microplastics, with limited differences between low- and high-visitation periods. This pattern may be related to its location next to rental cabins, where greater control and cleaning may occur. Carbonaceous particles were not included in the confirmed microplastic count because FTIR did not confirm a synthetic polymeric composition; therefore, they are discussed separately as anthropogenic carbonaceous residues potentially associated with beach cooking, bonfires, or combustion-derived inputs.

Q2 and Q3 showed higher particle counts than Q1 and stronger short-term variability in morphology. Confirmed fibres and films increased during the high-visitation period, while foams and fragments remained relevant components of the recovered confirmed microplastic fraction. Separately recorded carbonaceous residues were more frequent during the high-visitation period, which may reflect combustion-related recreational activities such as cooking or bonfires, but these particles were not interpreted as microplastics without polymer confirmation.

When aggregated across sectors and excluding non-confirmed carbonaceous particles, the low-visitation period was characterized by a predominance of foam and fragments, whereas the high-visitation period showed a higher relative contribution of films and fibres. Confirmed large microplastic abundance changed only slightly between the two campaigns, indicating that compositional differences were more evident than changes in total confirmed particle abundance.

These short-term differences suggest that visitor pressure may influence the type of debris and particle morphologies observed on the beach, but the present design does not support a robust seasonal or causal attribution. Large-microplastic occurrence may also reflect hydrodynamic sorting, wind transport, wave action, tidal redistribution, rainfall, prior cleaning, and fluvial inputs from the nearby Grijalva River. Therefore, tourism-related inputs should be interpreted as one component of a multi-source coastal transport system rather than as the sole driver.

Moreover, fragmentation dynamics driven by photo-oxidative weathering and abrasion processes further contribute to morphological transformation of plastics in coastal environments (Andrady, 2011).

### FTIR spectroscopy

**Fig. 4:**
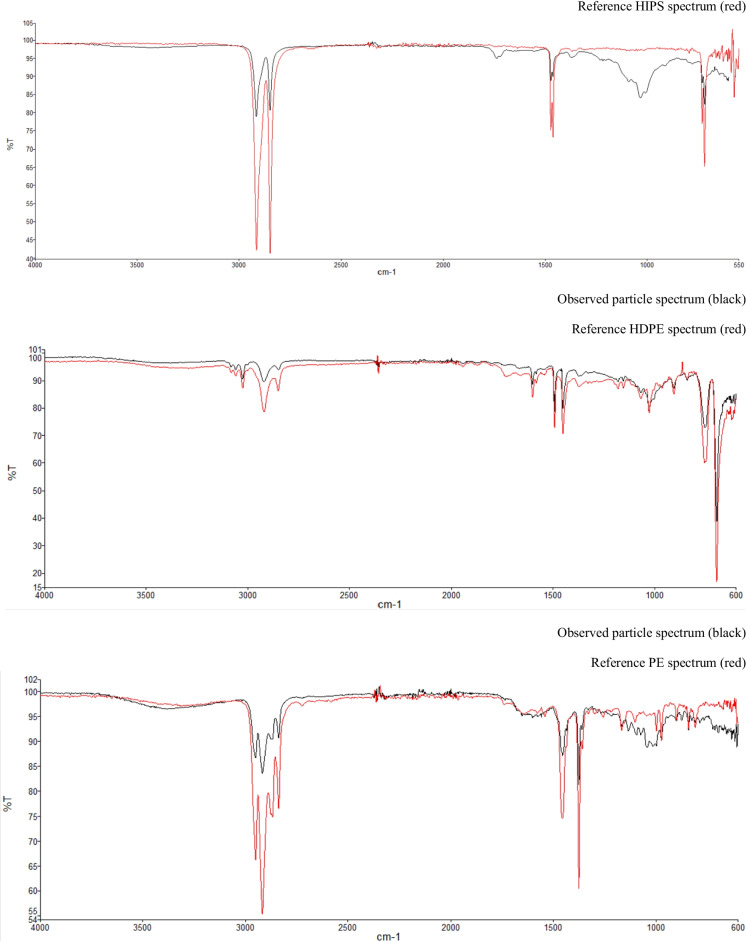
Representative FTIR spectra of foam, fragment, and fibre particles recovered from Playa Azul beach sediments. Polymer identification was performed by comparing the environmental particle spectrum (black line) with the corresponding reference polymer spectrum (red line) shown in each panel. The dominant polymers identified were high-impact polystyrene (HIPS) in foams, high-density polyethylene (HDPE) in fragments, and polyethylene (PE) in fibres. The *x*-axis represents wavenumber (cm^−1^), the standard FTIR unit describing the number of wave cycles per centimetre

Figure 4 presents the FTIR spectra obtained from representative particles. The majority of the analysed foams were identified as high-impact polystyrene (HIPS), with similarity indices ranging from 95 to 98% relative to reference spectra in the instrument library. Fragments showed match values between 90 and 95% with high-density polyethylene (HDPE), while fibres exhibited similarity levels of 90 to 92% with PE, which demonstrates a high correspondence with materials commonly used in packaging and rigid plastic products.

In the case of the fibres, the analysis showed similarity percentages of 90 to 92% with polyethylene, PE, which suggests a possible origin from synthetic textiles, cords, or plastic components associated with recreational activities. The rest of the particles analysed showed lower match values, attributable to the advanced degree of deterioration, weathering-induced oxidation, or the small size of the MPs, factors that make precise identification of the base polymer difficult. These polymer profiles are consistent with the dominant sources in coastal recreational environments: HIPS/foam from food containers and packaging, HDPE from rigid containers and caps, and PE from ropes/lines. The observed shift refers primarily to morphological composition. Polymer composition was assessed only for the FTIR-confirmed subset and therefore was used to support source interpretation, not to infer statistically robust polymer-level changes between visitation periods.

The observed distribution agrees with the literature documenting the prevalence of olefin polymers and PS on beaches due to their widespread use and persistence under surface weathering. Furthermore, the foam signal suggests direct contributions from disposable food items, while the fibres signal is consistent with recreational use and marine activities (Andrady, 2011).

Spectra with reduced matches are explained by photo-oxidation and abrasion processes that introduce bands (e.g. carbonyls) and decrease the signal-to-noise ratio in small or darkened particles, shifting the spectra relative to reference libraries. This effect, widely described for exposed plastics, can lead to under-assignment of polymers or to generic classifications when the optical mass is low or when fillers/pigments are present (Andrady, 2011; Song et al., 2015). In this sense, the similarity percentages reported here are reasonable for environmental matrices and support the interpretation that the “other” fraction includes highly degraded polymers and/or additive mixtures. The reliability of the assignment is reinforced by the coupling between morphology and polymer (foams → HIPS; rigid fragments → HDPE; fibres → PE), a pattern also observed in comparative evaluations of identification methods, where FTIR provides monitoring for > 300 µm and is complemented by µ-FTIR/Raman for small and dark particles (Song et al., 2015). Additionally, the use of curated and harmonized libraries reduces false positives and optimizes productivity across different laboratories; the literature even recommends specific libraries for weathered polymers and spectral pre-treatment protocols (Primpke et al., 2018). In short, the results confirm that, even in contexts of low overall abundance, the polymeric signature allows for the inference of dominant sources and guides management.

### Beach quality assessment

Using the corrected sampled area and confirmed large microplastic counts, MPPI values were treated as descriptive screening indicators for short-term comparison between campaigns. Because only two 1-week campaigns were available, the index should not be interpreted as a definitive long-term classification of beach pollution status.

Regarding compositional structure expressed through the CMPI, calculations were restricted to confirmed large microplastic morphologies. The low-visitation period was characterized by a predominance of foams and fragments, whereas the high-visitation period showed a higher relative contribution of films and fibres. Carbonaceous particles were excluded from CMPI because FTIR did not confirm a synthetic polymeric composition.

Thus, although overall confirmed large-microplastic abundance at Playa Azul remained low, the compositional profile exhibited short-term differences associated with contrasting visitation periods. These differences should be interpreted cautiously because the study design does not isolate tourism from hydrodynamic, meteorological, fluvial, or beach-cleaning effects.

This contrasts with the moderate to high MP abundances reported for beaches of the Colombian Caribbean and the Veracruz Reef System National Park, where higher loads reflect stronger anthropogenic pressure or less effective management (Peralta-Peláez et al., 2023; Rangel-Buitrago et al., 2021). Nevertheless, the findings align with global assessments identifying inadequate coastal waste management as a major driver of plastic entry into marine systems (Jambeck et al., 2015).

The divergence between low total abundance and marked compositional shifts indicates that beach quality responds rapidly to the intensity and nature of recreational use, including disposable consumption, textile shedding, and food preparation activities. Similar patterns have been described in tourist-dominated beaches, where visitor pressure modifies dominant debris morphologies without proportionally increasing total loads (Browne et al., 2011; Grelaud & Ziveri, 2020).

The predominance of foam is consistent with contributions from expanded PS food containers and beverage packaging, whose low density and fragility favour fragmentation and shoreline accumulation. The increase in fibres during the high-visitation period corresponds with greater textile use and recreational rope handling. Separately recorded carbonaceous residues may reflect beach cooking or bonfire activities during peak visitation, but they were not classified as microplastics without polymer confirmation. These transformations are further reinforced by photo-oxidative and abrasive weathering processes that convert larger plastic items into fragments and films, thereby modifying morphological signatures without necessarily increasing total mass (Andrady, 2011).

Overall, the results support a cautious interpretation in which visitor pressure is clearly associated with increased total beach debris. Although increased visitation plausibly contributed to the observed rise in beach debris, large-microplastic occurrence may also reflect hydrodynamic sorting, wind transport, wave action, tidal redistribution, and fluvial inputs from the nearby Grijalva River. Therefore, tourism-associated inputs should be interpreted as one component within a more complex coastal transport system. Furthermore, confirmed large-microplastic abundance and morphology showed short-term descriptive variation that requires broader temporal and spatial replication before robust long-term conclusions can be drawn.

### Limitations and future monitoring needs

The study provides a short-term baseline comparison between two contrasting visitation periods and should not be interpreted as a complete seasonal or interannual assessment. The sampling design included two 1-week campaigns and therefore does not capture interannual variability, complete climatic seasonality, hydrodynamic cycles, or episodic inputs from rainfall, river discharge, tides, waves, and wind-driven transport. The results show that total beach debris increased with visitor numbers during the high-visitation period. In contrast, changes in confirmed large microplastic abundance and morphology should be interpreted cautiously because the short-term design does not isolate tourism from other potential drivers such as wind, waves, tides, rainfall, river discharge, sediment redistribution, or prior beach-cleaning activities. Consequently, tourism is interpreted as a plausible contributing factor, particularly for total beach debris, but not as an isolated causal driver of large-microplastic abundance or composition.

Because the analytical workflow focused on particles retained above 0.84 mm, the results do not represent total microplastic abundance. Smaller fractions may be substantially underestimated. Furthermore, MPPI and CMPI should be interpreted as descriptive screening indicators rather than definitive long-term environmental quality classifications.

## Conclusions

The results show that total beach debris increased markedly with visitor numbers during the high-visitation period at Playa Azul. In contrast, changes in confirmed large-microplastic abundance and morphology should be interpreted cautiously because the short-term design does not isolate tourism from other potential drivers such as wind, waves, tides, rainfall, river discharge, sediment redistribution, or prior beach-cleaning activities. Morphological and polymer characterization indicated the predominance of HIPS foams, HDPE fragments, and PE fibres, supporting the relevance of disposable food items, packaging, and recreational textiles as plausible sources. Carbonaceous particles were recorded separately and were not classified as confirmed microplastics because their polymeric origin was not confirmed by FTIR. Future monitoring should expand the temporal and spatial scale of sampling, include additional beach sectors and climatic periods, and incorporate complementary matrices such as the water column and biota. Such an expanded design would provide a more comprehensive assessment of plastic contamination in Tabasco coastal areas and would support more robust MPPI and CMPI time series for comparison with other coastlines. These improvements are necessary to guide prevention, reduction, reuse, and recycling strategies for marine debris and to strengthen evidence-based coastal management policies.

## Data Availability

The datasets generated during and/or analysed during the current study are available from the corresponding author on reasonable request.
